# MAP2 phosphorylation: mechanisms, functional consequences, and emerging insights

**DOI:** 10.3389/fncel.2025.1610371

**Published:** 2025-07-30

**Authors:** Jiali Lyu, Andrew G. DeMarco, Robert A. Sweet, Melanie J. Grubisha

**Affiliations:** ^1^School of Medicine, Tsinghua Medicine, Tsinghua University, Beijing, China; ^2^Translational Neuroscience Program, Department of Psychiatry, University of Pittsburgh School of Medicine, Pittsburgh, PA, United States; ^3^Department of Psychiatry, University of Pittsburgh School of Medicine, Pittsburgh, PA, United States; ^4^Department of Neurology, University of Pittsburgh School of Medicine, Pittsburgh, PA, United States

**Keywords:** microtubule-associated protein 2 (MAP2), phosphorylation, kinase, cytoskeleton, microtubule, dendrite

## Abstract

Microtubule-associated protein 2 (MAP2) is a key regulator of cytoskeletal dynamics and neuronal function. It stabilizes microtubules, shapes dendrites, influences synaptic plasticity, and regulates transportation and protein synthesis through its interactions with other proteins. MAP2 undergoes extensive phosphorylation, which dynamically modulates these interactions and alters MAP2 functions. This review provides a comprehensive overview of MAP2 structure, its diverse functional roles in neurons, the kinases that regulate its phosphorylation. We highlight how phosphorylation by Src family kinases, proline-directed kinases, MARK, PKA, PKC, and CAMKII governs MAP2’s role in cytoskeletal organization, protein chaperone activity, and dendrite outgrowth.

## 1 Introduction

The regulation of neuronal structure and function is critical for understanding the mechanisms underlying neurodevelopmental and neurodegenerative disorders. Microtubules are a key component of the neuronal cytoskeleton that provide structural support and facilitate intracellular trafficking, both of which are essential for maintaining neuronal architecture and synaptic function (Goodson and Jonasson, 2018). The stability and organization of the microtubule network are regulated by microtubule-associated proteins (MAPs), which play an integral role in modulating microtubule dynamics (Dehmelt and Halpain, 2004). Among these, microtubule-associated protein 2 (MAP2) is the primary dendritic MAP, playing a pivotal role in dendrite formation and organization by cross-linking and stabilizing microtubules (Dehmelt and Halpain, 2004). The specific localization of MAP2 in dendrites has underscored its importance in synaptic plasticity and neuronal signaling, though new functions of MAP2 continue to be identified (Abel et al., 1997; Gumy et al., 2017; Ribeiro and de Wit, 2017; Harada et al., 2002; Ainsztein and Purich, 1994; Grubisha et al., 2021; Kim et al., 2020).

MAP2 is highly phosphorylated *in vivo*, and its phosphorylation state is tightly regulated during neuronal development by multiple signaling pathways (Yang et al., 2023; Riederer et al., 1995). Phosphorylation influences MAP2’s interactions with other proteins, especially its interaction with microtubules and its ability to stabilize them (Grubisha et al., 2021; Brugg and Matus, 1991; Komulainen et al., 2014; Liu et al., 2019; Zamora-Leon et al., 2001; DeGiosio et al., 2023). Dysregulated MAP2 phosphorylation has been implicated in a variety of neurological and psychiatric disorders, including Alzheimer’s disease and schizophrenia, highlighting the importance of understanding how phosphorylation modulates MAP2’s function (Grubisha et al., 2021; DeGiosio et al., 2019; Zhang and Dong, 2012; Rudrabhatla et al., 2011). Despite significant advances in research, the precise mechanisms by which phosphorylation alters MAP2 activity, and the extent to which these changes contribute to disease progression, remain incompletely understood.

This review aims to provide a comprehensive view of current knowledge of MAP2 phosphorylation and its impact on MAP2 affinity to its interactors. We will discuss the functional consequences of phosphorylation at key sites. Additionally, we will highlight recent findings on the kinases that regulate MAP2 phosphorylation.

## 2 MAP2 structure

**Figure 1:**
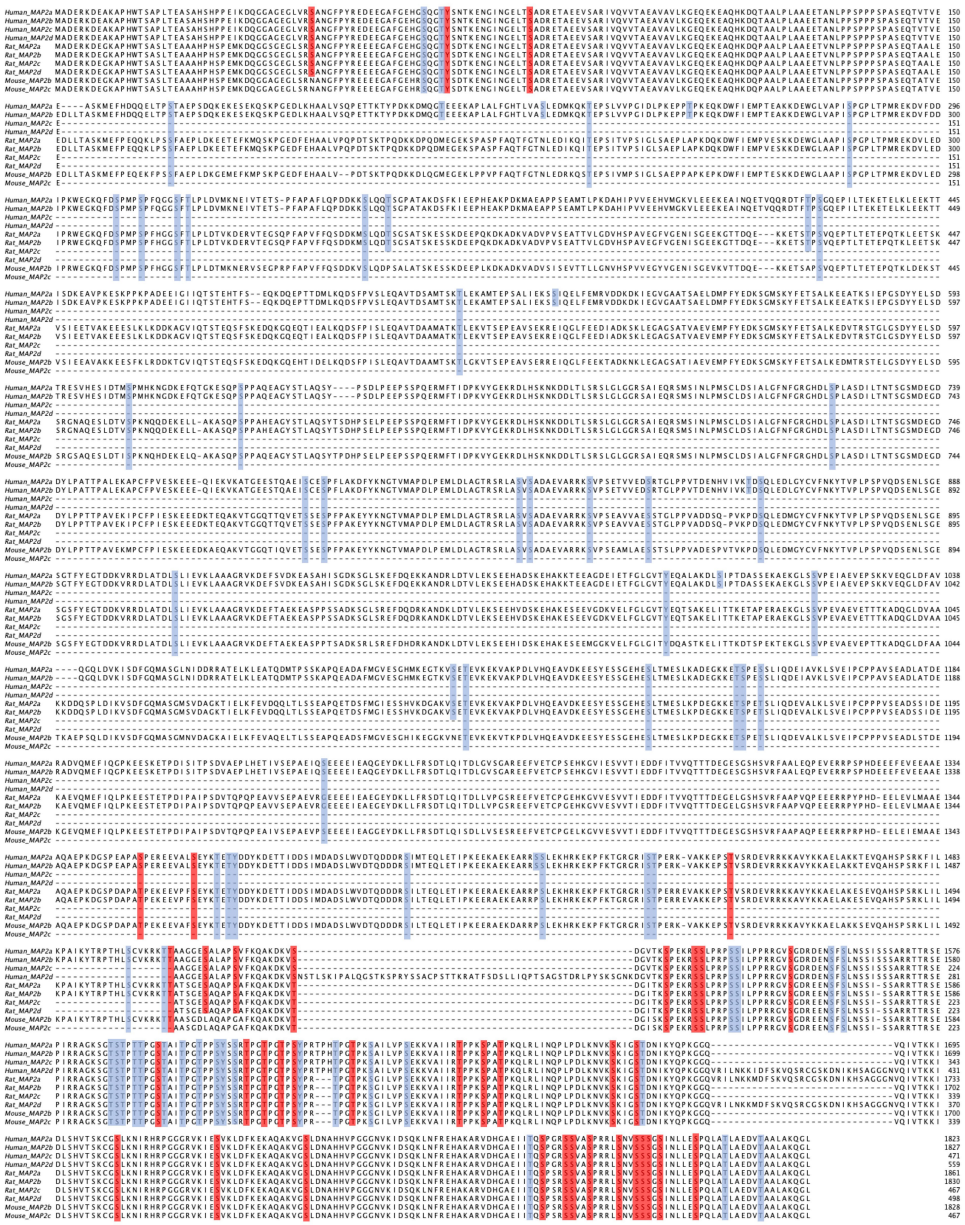
Multiple sequence alignment of MAP2 isoforms from human, mouse, and rat. Conserved regions are evident at the N-and C-termini, while notable differences are seen in the central region corresponding to the presence or absence of the projection domain. Residues highlighted in blue represent high-throughput screened phosphorylation sites, whereas those in red indicate low-throughput validated phosphorylation sites. Note, however, that many residues conserved across species are nevertheless identified by slightly different amino acid positions (e.g., see S1782 in human MAP2b, S1785 in rat MAP2b, and S1783 in mouse MAP2b). The alignment was performed using MAFFT and visualized with Jalview (Katoh and Standley, 2013; Waterhouse et al., 2009).

**Figure 2:**
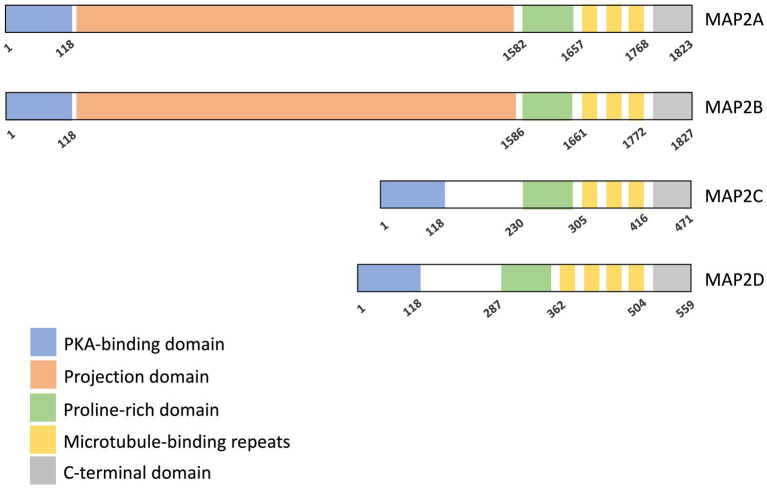
Domain organization of MAP2 isoforms. Four isoforms of human MAP2 are shown here. All isoforms share a PKA-binding domain, a proline-rich domain, a microtubule-binding domain (MTBD), and a C-terminal region. MAP2A and MAP2B additionally contain a projection domain. Start and end amino acid positions for each domain are indicated (Melkova et al., 2019). Microtubule interaction is primarily mediated by the microtubule-binding repeats, with contributions from the proline-rich and C-terminal domains. The projection domain is known to modulate the affinity of MAP2 for microtubules.

MAP2 is produced in several isoforms through alternative splicing, which can be divided into two major groups: high-molecular-weight (HMW) isoforms, MAP2A (280 kDa) and MAP2b (270 kDa), and low-molecular-weight (LMW) isoforms, MAP2c (70 kDa) and MAP2d (75 kDa) (Figures 1, 2) (Neve et al., 1986; Kalcheva et al., 1995). Both HMW and LMW isoforms share four core domains: a PKA-binding domain, a proline-rich domain, a microtubule-binding domain, and a C-terminal domain. The HMW variants are distinguished by the presence of an additional projection domain located between the PKA-binding and proline-rich domain, which is absent in the LMW variants (Figure 2) (Kindler et al., 1990).

The microtubule-binding domain contains three or four conserved binding repeats, each 18 amino acids in length. It is critical for MAP2’s primary function of binding and stabilizing microtubules (Doll et al., 1993; Goode et al., 1997). Other regions, including the proline-rich and the C-terminal domain, also contribute to this binding activity (Chen et al., 1992; Ferralli et al., 1994). The projection domain, exclusive to HMW isoforms, acts as a regulator of microtubule interactions and also a spacer to ensure adequate spacing between microtubules and nearby cellular components (Chen et al., 1992). Furthermore, it prevents the entry of MAP2A and MAP2B into axons, thereby directing their localization to dendrites (Kanai and Hirokawa, 1995).

MAP2 isoforms are intrinsically disordered in their unbound state, characterized by a lack of secondary structure and a high degree of conformational flexibility. This disordered nature allows MAP2 to interact dynamically with the complex cytoskeleton (Kosciolek et al., 2017; Novacek et al., 2013). Importantly, this intrinsic disorder can be modulated by post-translational modifications, such as phosphorylation, which can alter MAP2’s interactions and functional properties within the cell (Grubisha et al., 2021; Jansen et al., 2017). Despite this disordered nature, MAP2 also contains rigid motifs, including the PKA-binding and microtubule-binding motifs (Melkova et al., 2019).

## 3 MAP2 functions

Knowledge of MAP2 functions provides the context for understanding the significance of its phosphorylation. As a key regulator of cytoskeletal dynamics, cargo transport, and dendritic signaling, MAP2’s functions are intricately linked to its structural and interaction properties. Phosphorylation plays a pivotal role in modulating these functions by altering MAP2’s interactions with cytoskeletal components and other proteins.

### 3.1 Cytoskeleton structure

MAP2 was identified as a microtubule-associated protein due to its ability to bind to and stabilize microtubules. It exerts this stabilization effect by reducing the frequency of catastrophes and slowing down the shortening rate of microtubules (Gamblin et al., 1996; Itoh and Hotani, 1994). Furthermore, MAP2 can facilitate microtubule bundling, acting as a spacer within the microtubule bundle (Chen et al., 1992; Weisshaar and Matus, 1993). MAP2 has been shown to promote tubulin polymerization through its microtubulin-binding region, which can be regulated by phosphorylation (Ainsztein and Purich, 1994). MAP2, via its microtubulin-binding region can also bind to other cytoskeleton components, such as actin, acting as the crosslinker between microtubule and actin. MAP2 can also interact with Plectin (an intermediate filament-associated cytolinker protein), which can antagonize the microtubule stabilization ability of MAP2 (Valencia et al., 2013).

### 3.2 Dendritic morphology

Neurite initiation depends on the rapid reorganization of the cytoskeleton, achieved through the coordination of microtubules and actin filaments. Given its ability to influence both microtubule and actin organization, it creates a conducive environment for triggering neurite initiation (L. Dehmelt et al., 2003). MAP2 has been shown to be shifted and concentrated in the outgrowth and branching areas of dendrites in several cell lines (Matus et al., 1986; Ferreira et al., 1989; Chamak et al., 1987). Evidence indicates that the expression of MAP2c can induce the rapid formation of dense and stable microtubule bundles at the distal plus end. Conversely, knocking down MAP2 in primary cortical neurons and hippocampal neurons results in a reduction in both the number and length of dendrites, as well as a decrease in microtubule presence within dendrites (Sharma et al., 1994; Harada et al., 2002; LeClerc et al., 1993; Dehmelt et al., 2003). MAP2 serves as the receptor for neurosteroid pregnenolone (PREG) to enhance neurite outgrowth by stimulating microtubule polymerization (Fontaine-Lenoir et al., 2006).

### 3.3 Synaptic plasticity

MAP2 is essential for long-term potentiation (LTP) and the dendritic spine changes induced by LTP. Kim et al. have demonstrated that MAP2 is translocated to the spines in response to LTP stimulation and the knockdown of HMW MAP2 causes a deficit of LTP induction and abolishes LTP-induced surface delivery of AMPA receptors and spine enlargement. However, the knockdown of LMW MAP2 does not show the same effect (Kim et al., 2020). Also, MAP2 contributes to the inhibition of microtubule entry into dendritic spines induced by long-term depression (LTD) by mediating the prolonged redistribution of EB3 (End-binding protein 3) along MAP2-positive microtubule bundles in the dendritic shaft, thereby suppressing microtubule dynamics (Kapitein et al., 2011). EB3 is a member of microtubule plus-end binding proteins, which regulate microtubule dynamics (Jaworski et al., 2009).

### 3.4 Protein folding

MAP2 has been reported to show a chaperone-like activity, preventing protein aggregation under thermal or chemical induction and assisting in enzyme refolding (Sarkar et al., 2004). Specifically, MAP2c plays a role in preventing tau aggregation, which is implicated in Alzheimer’s disease. Electron microscopy studies show that MAP2c inhibits arachidonic acid-induced tau aggregation *in vitro*, suggesting a role in maintaining tau homeostasis. Despite the high homology between the C-terminal regions of tau and MAP2c, the N-terminal region of MAP2c alone does not mediate its chaperone activity (Mitra et al., 2015).

### 3.5 Cargo transportation

MAP2 is responsible for regulating kinesin/dynein-dependent microtubule transporting in neurons (Heins et al., 1991; Lopez and Sheetz, 1993; Seitz et al., 2002; Hagiwara et al., 1994). This regulation is achieved in two ways: (1) MAP2 and motor proteins both bind to the C-terminus of tubulin, competing for the same binding sites on microtubules; (2) HMW MAP2 can interact with motor proteins, like KIF5B, preventing its binding to microtubules through the steric inhibition caused by MAP2B’s projection domain. By coordinating with motor proteins, MAP2B forms a filtering zone at the axon initial segment that controls cargo entrance to the axon in sensory neurons (Hagiwara et al., 1994; Gumy et al., 2017). The regulation by MAP2 of cargo transportation thus enables the polarized distribution of neuronal proteins and organelles, which is vital to normal neuronal function.

### 3.6 Signal transduction

MAP2 serves as the dominant anchoring protein of cAMP-dependent protein kinase (PKA) in dendrites, whose kinase activity is known to be involved in various biological functions in neurons (Abel et al., 1997; Malleret et al., 2001). MAP2 binds to the regulatory subunit II (RII) of PKA through its N-terminus and determines the localization of PKA in neurons (Vallee et al., 1981; Theurkauf and Vallee, 1982). MAP2 serves as a reservoir for PKA. A deficit in MAP2 impairs the ability of PKA to phosphorylate key substrates, such as CREB and AMPA receptors, following signal stimulation. This disruption ultimately blocks the downstream signal transduction pathway (Zhong et al., 2009; Harada et al., 2002). Furthermore, the RII subunit of PKA, but not RI, binds specifically to MAP2 and is shielded from calpain degradation when associated with MAP2 (Alexa et al., 1996).

Additionally, MAP2 comprises 11 PXXP motifs which are the potential binding ligands of Src homology 3 (SH3) domains. MAP2 interacts with several SH3 domain-containing proteins, for example, the non-receptor protein tyrosine kinase Src and Fyn and the adaptor protein Grb2 (see 4.1 below). Src family kinases are a group of non-receptor kinases containing conserved SH3 and SH2 domains, which mediate substrate recognition and protein–protein interactions (Parsons and Parsons, 2004). These kinases play critical roles in neuronal signaling and cytoskeletal regulation. Also, the MAP2 Co-IP experiment identified several SH-domain-containing proteins within the MAP2 interactome, including guanine nucleotide exchange factors and members of the Rho family of GTPases, both of which are involved in signal transduction (Lyu et al., 2024). The interaction between MAP2 and proteins mentioned above may indicate the role of MAP2 as a scaffold protein.

### 3.7 mRNA binding and protein synthesis

MAP2 has been implicated in the regulation of protein synthesis. Overexpression of the MAP2c isoform has been shown to inhibit protein synthesis in HEK cells, suggesting a direct role in translational control, though the mechanism is still unclear (Grubisha et al., 2021). This inhibitory role is further supported by the composition of the MAP2 interactome, which includes multiple RNA-binding proteins known to modulate translation, many of which are associated with suppressing protein synthesis. A key interaction underlying this regulatory function is between MAP2 and insulin-like growth factor 2 mRNA-binding protein 1 (IMP1). MAP2 binds directly to IMP1 via its K-homology (KH) domains, which are critical for nucleic acid binding (Nielsen et al., 2002). Furthermore, proteomic analyses of the MAP2 interactome have identified numerous RNA-binding proteins involved in the regulation of translation, many of which are associated with inhibitory effects on protein synthesis (Grubisha et al., 2021). Together, these findings suggest that MAP2 contributes to the modulation of protein synthesis, potentially through interactions with RNA-binding proteins and direct effects on translational machinery.

## 4 Key kinases regulating MAP2 phosphorylation

MAP2 undergoes extensive phosphorylation *in vivo*, which, as mentioned earlier, is crucial due to its intrinsically disordered nature. This phosphorylation can induce significant changes in its structure and thereby influencing its function primarily by regulating its interactions with other proteins (Tsuyama et al., 1987; Newcombe et al., 2022; Melkova et al., 2019). Phosphorylation plays a crucial role in regulating MAP2 function, primarily by modulating its interactions with other proteins. Over the years, considerable research has focused on identifying the specific phosphorylation sites on MAP2, as well as elucidating how phosphorylation modulates its activities. These modifications, catalyzed by a range of kinases, influence MAP2’s role in cytoskeletal dynamics, transport, and chaperone activity. Understanding how these kinases regulate MAP2 phosphorylation is essential for elucidating MAP2’s involvement in neuronal development and disease.

### 4.1 Src family kinase

MAP2 contains a proline-rich RTPPKSP motif which specifically interacts with the SH3 domain of Src family kinases. Among the Src family kinases, MAP2 has been reported to interact with two key members of the Src family kinase, Fyn and Src (Zamora-Leon et al., 2001; Sontag et al., 2012). Phosphorylation of MAP2 by Fyn was initially demonstrated *in vitro* (Zamora-Leon et al., 2001), with mutagenesis studies of MAP2c later identifying tyrosine 67 (Y67) as the primary phosphorylation site on the MAP2c isoform (Zamora-Leon et al., 2005). Phosphorylation at this site has also been confirmed in the human fetal brain, and it assists in recruiting the SH2 domain of Grb2 (Zamora-Leon et al., 2005). The binding of Fyn to MAP2 also inhibits the interaction between MAP2 and protein phosphatase 2A (PP2A), thereby suppressing PP2A’s dephosphorylation activity, which may stabilize MAP2 phosphorylation states (Sontag et al., 2012).

Interestingly, although MAP2 interacts with Src via the SH3 domain, Src does not phosphorylate MAP2 *in vivo* (Lim and Halpain, 2000).

### 4.2 Proline-directed protein kinases (PDPK)

PDPKs are a class of serine/threonine kinases that specifically phosphorylate substrates at serine or threonine residues followed by a proline (Ser/Thr-Pro motifs). These kinases are crucial regulators of cell signaling pathways. The MAPK (mitogen-activated protein kinase) family, cyclin-dependent kinases (CDKs), and glycogen synthase kinase 3 (GSK-3) are among the well-known PDPKs. MAP2’s proline-rich domain is abundant in Ser/Thr-Pro motifs, making it a target for PDPKs.

#### 4.2.1 Mitogen-activated protein kinases (MAPKs)

The family of mitogen-activated protein kinases (MAPKs) includes extracellular signal-regulated kinase (ERK), p38, and c-Jun NH (2)-terminal kinase (JNK). Among them, ERK and JNK are known kinases of MAP2. ERK1 and ERK2 are coded by two distinct genes, MAPK3 and MAPK1,and are highly expressed in the brain. They are associated with microtubules and levels increase during brain development (Busca et al., 2016; Boulton et al., 1991; Reszka et al., 1995). ERK is a key component of the Raf–MEK–ERK signaling pathway, contributing to cellular processes like adhesion, migration, and neuronal differentiation (Busca et al., 2016). The phosphorylation sites of ERK in MAP2c have been screened by NMR spectrometry with single residue resolution (Plucarova et al., 2022), identifying 12 Pro-Xxx-Ser/Thr-Pro sequences and 7 Ser/Thr-Pro motifs of MAP2c as the targets of ERK2 (Plucarova et al., 2022). Whether, or to what extent, these sites may also be targets for regulation by ERK1 is not established. Phosphorylation of MAP2 by ERK happens primarily in the proline-rich domain and microtubule-binding domain in MAP2, and it disrupts the interaction of MAP2 with various proteins, namely regulatory subunit RIIα of cAMP-dependent PKA, Grb2, Fyn, Src, and even ERK itself (Lim and Halpain, 2000; Zamora-Leon et al., 2001; Plucarova et al., 2022; Hoshi et al., 1992). Also, the phosphorylation by ERK abolishes the ability to induce tubulin nucleation and polymerization of MAP2 *in vitro* (Hoshi et al., 1992). During dendrite formation induced by neuronal depolarization, ERK and CAMKII activation enhances MAP2 phosphorylation and expression, highlighting ERK’s role in activity-dependent neuronal remodeling (Vaillant et al., 2002). Also, phosphorylation of MAP2 by ERK is crucial for its translocation to dendritic spines in response to LTP stimulation (Kim et al., 2020).

The function of phosphorylation by ERK2 at specific sites has also been studied. Specific phosphorylation events, such as those at T197 and T293 of MAP2c, further impair microtubule assembly and inhibit MAP2’s actin-binding capacity, linking ERK signaling to cytoskeletal reorganization (DeGiosio et al., 2023). It should be pointed out that ERK2 phosphorylates S422 of rat MAP2c (homologous to S426 of human MAP2c and S1782 of human MAP2b). Notably, S426/S1782 is abnormally hyperphosphorylated in schizophrenia. Phosphomimetic mutation at this site has been shown to reduce dendritic length, complexity, and spine density in mouse brains. Co-immunoprecipitation studies reveal that phosphorylation at S1782 disrupts interactions with a significant portion of the MAP2 interactome, including tubulin and actin, while promoting binding to a smaller subset of proteins, including protein phosphatase 1 L (PPM1L) and Kelch-Like Family Member 8 (KLHL8) (Grubisha et al., 2021; Lyu et al., 2024; DeGiosio et al., 2023).

JNKs, are critical regulators of cellular responses to stress, inflammation, and synaptic plasticity (Schellino et al., 2019). JNK activation is a hallmark of pathological cell death in conditions such as Alzheimer’s disease, emphasizing its role in neuronal dysfunction (Yarza et al., 2015). MAP2 was first identified as a downstream effector of JNK in neurons in 2005 (Bjorkblom et al., 2005). It’s reported that JNK can phosphorylate MAP2 in intact neurons and the phosphorylation sites accumulate in the proline-rich domain of MAP2 (Bjorkblom et al., 2005). Phosphorylation by JNK promotes MAP2-dependent process elongation, facilitating dendrite shape regulation in neurons. Notably, JNK-mediated regulation of dendrite morphology is dominant over ERK in this context (Bjorkblom et al., 2005). Mechanistically, JNK1 phosphorylates high-molecular-weight MAP2 (HMW-MAP2) at T1619, T1622, and T1625, enhancing MAP2’s interaction with and stabilization of microtubules (Komulainen et al., 2014).

#### 4.2.2 Glycogen synthase kinase 3 (GSK3)

GSK is a serine/threonine kinase implicated in diverse cellular processes, including metabolism, cytoskeletal regulation, and neuronal function. In mammals, GSK3 exists in two highly homologous isoforms, GSK3α and GSK3*β*, encoded by two different genes (Woodgett, 1990). GSK3 plays a broad role in neurodevelopment, particularly through its regulation of a wide range of transcription factors and cytoskeletal dynamics, including microtubules (Hur and Zhou, 2010). The regulation of the cytoskeleton by GSK3 is in part by GSK3-mediated phosphorylation of MAPs, like tau, MAP1B, and MAP2 (Barnes et al., 2007; Trivedi et al., 2005; Sanchez et al., 2000). As with many other PDPKs, GSK3 phosphorylates MAP2 within its proline-rich domain, targeting specific threonine residues (Sanchez et al., 1996). Initial studies identified phosphorylation of GSK3α/β at the threonine residues within the sequence “RTPGTPGTPSY,” which was also detected *in vivo* in rat brain (Sanchez et al., 1996). Further investigations localized these phosphorylation events to T1620 and T1623 in the MAP2B isoform (Sanchez et al., 1996). Functional studies in COS-1 cells revealed that GSK3β-mediated phosphorylation of MAP2C reduced its binding affinity for microtubules, disrupting the formation of microtubule bundles and thereby altering cytoskeletal organization (Sanchez et al., 2000).

The phosphorylation activity of GSK3 is tightly regulated and can be reversed by protein phosphatases, specifically protein phosphatase 1 (PP1) and 2A (PP2A) (Sanchez et al., 1996). Pathologically, MAP2 phosphorylated at T1620 and T1623 has been observed within granules formed during the early stages of neurofibrillary tangle formation, a hallmark of neurodegenerative diseases. Notably, these granules also contain hyperphosphorylated GSK3β (Andres-Benito et al., 2023). Additionally, evidence suggests that activation of the Akt/GSK3 pathway is associated with increased MAP2 expression, though the underlying mechanisms remain unclear (Chang et al., 2013; Repar et al., 2018; Zhang et al., 2024).

#### 4.2.3 Cyclin-dependent kinases (CDKs)

Cyclin-dependent kinases (CDKs) are a family of serine/threonine kinases crucial for cell cycle regulation. Beyond their well-established roles in cell cycle progression, CDKs are also implicated in transcriptional control and neuronal functions (Lim and Kaldis, 2013; Dhariwala and Rajadhyaksha, 2008). CDKs require association with cyclins or other regulatory proteins for activation, and their activity is tightly controlled by phosphorylation (Lim and Kaldis, 2013; Dhariwala and Rajadhyaksha, 2008). In the nervous system, CDK5 is the most abundant CDK in neurons. CDK5 does not regulate the cell cycle like other CDKs but instead plays a pivotal role in neuron development and functions (Dhariwala and Rajadhyaksha, 2008). It modulates cytoskeletal proteins like MAP2 and tau, influencing neuronal structure and connectivity (Shah and Lahiri, 2017).

The relation of MAP2 and CDK5 was first observed in mice with NPC-1 gene mutation, where MAP2 was found to be hyperphosphorylated and accumulated in the brain. This coincided with increased CDK5 activity and elevated levels of its activators, p25 and p35 (Bu et al., 2002). Further studies revealed that CDK5 phosphorylates HMW MAP2 at multiple sites, including threonine _1613_TPGTPGTP_1620_ and S1782 (homologous to S426 in LMW MAP2, discussed earlier). The phosphorylation within the _1613_TPGTPGTP_1620_ motif, which contains two PXXP sequences, is predicted to interfere with interactions involving SH3 domains (Tseng et al., 2005). Phosphoproteomic studies using cdk5^−/−^ mouse brains further identified hypophosphorylation of MAP2 at T1650, suggesting CDK5’s site-specificity in regulating MAP2 phosphorylation (Contreras-Vallejos et al., 2014).

Other than CDK5, MAP2 can be phosphorylated by CDK1 (cdc2 kinase) and CDK2. 60% of CDK1 phosphorylation events are localized to the microtubule-binding domain (Itoh et al., 1997). This includes phosphorylation of the “RTPGTPGTPSY” sequence, which is known to impair MAP2’s microtubule nucleation and stabilization activity (Sanchez et al., 1996; Itoh et al., 1997). CDK2, on the other hand, phosphorylates MAP2C at S264, S178, and other ERK2-targeted sites except for S274 and S448, with enhanced efficiency at S178 (Plucarova et al., 2022).

### 4.3 Microtubule affinity-regulating kinases (MARKs)

Microtubule affinity-regulating kinases (MARKs), also known as Par-1 kinases, are serine/threonine kinases that regulate microtubule dynamics by phosphorylating microtubule-associated proteins (MAPs), including MAP2, MAP4, and tau (Drewes et al., 1997). MARKs are involved in maintaining cytoskeletal stability, neuronal polarity, and intracellular transport, making them essential for proper neuronal function (Drewes et al., 1997).

Microtubule affinity-regulating kinases (MARKs) were first identified for their ability to phosphorylate microtubule-associated proteins (MAPs) and disrupt their interaction with microtubules (MTs), profoundly altering cytoskeletal dynamics (Drewes et al., 1995). The first discovered MARK isoform, p110^MARK^, was initially characterized for phosphorylating tau and later shown to phosphorylate MAP2 at conserved KXGS motifs in the MAP2 MT binding domain (Drewes et al., 1995). Two major phosphorylation sites in MAP2 targeted by p110^MARK^ are Ser1713 (KCGS motif) and Ser1682 (KIGS motif). This phosphorylation dissociates MAP2 from MTs, leading to increased dynamic instability of the microtubule network (Illenberger et al., 1996).

Further studies demonstrated that MARK1 and MARK2, primarily expressed in brain, share this ability to efficiently block MAP2-MT interactions (Drewes et al., 1997). Overexpression of MARK1 or MARK2 in CHO cells results in the destruction of the microtubule network without affecting the microfilament network, highlighting their specific regulatory role. Conversely, co-transfection with a MAP2c mutant (S319A/S350A) resistant to MARK phosphorylation effectively counteracted this microtubule destabilization, underscoring the direct correlation between KXGS motif phosphorylation and cytoskeletal disassembly (Drewes et al., 1997).

### 4.4 cAMP-dependent protein kinase (PKA)

PKA, a serine/threonine kinase activated by cyclic AMP (cAMP), plays a critical role in regulating cytoskeletal dynamics and synaptic plasticity (Huang et al., 2013). MAP2 serves as a major substrate of PKA in neurons (Theurkauf and Vallee, 1982, 1983). The regulatory subunit RII of PKA interacts with a conserved 83-amino-acid sequence at the distal N-terminus of MAP2 isoforms (MAP2A, MAP2B, and MAP2C) (Obar et al., 1989). *In vitro* studies demonstrate that PKA can phosphorylate MAP2B at up to 15 moles of phosphate per molecule, with 70% of these phosphorylation events distributed across the proline-rich and microtubule-binding domains (Itoh et al., 1997). Detailed analyses using tryptic digestion and two-dimensional phosphopeptide mapping identified 11 major PKA-specific phosphorylation sites, all localized to serine residues (Goldenring et al., 1985). These phosphopeptides were also present in the rat brain and GH3 cells (Diaz-Nido et al., 1990; Jefferson and Schulman, 1991). NMR spectrometry has further resolved these phosphorylation sites on MAP2C with single-residue precision (Jansen et al., 2017). Notably, four PKA phosphorylation sites overlap with those targeted by calcium/calmodulin-dependent protein kinase II (CaMKII) (Walaas and Nairn, 1989). During development, in cytosol, PKA phosphorylation of MAP2 increased from 2 days to adult in proportion to the increase in the concentration of MAP2 in chicken brains (Koszka et al., 1991).

Phosphorylation of MAP2 by PKA alters its interactions with microtubules and other cellular structures. PKA-mediated phosphorylation of MAP2 reduces its ability to bind to and nucleate microtubules, although its ability to stabilize microtubules remains unaffected (Itoh et al., 1997). Within the microtubule-binding domain of MAP2C, PKA phosphorylates serine residues at the KXGS motifs (S319, S350, and S382). These phosphorylations result in MAP2C dissociation from microtubules and redistribution to the peripheral actin cytoskeleton (Ozer and Halpain, 2000). Additionally, phosphorylation by PKA enhances MAP2C’s affinity for 14–3-3ζ, a regulatory protein that competes with tubulin for MAP2 binding, although this interaction does not disrupt MAP2C-microtubule binding (Jansen et al., 2017). PKA-mediated phosphorylation also abolished the chaperone-like ability of MAP2c for preventing tau fibril formation induced by arachidonic acid *in vitro* (Mitra et al., 2015).

The functional implications of PKA-mediated phosphorylation extend beyond microtubule dynamics. For example, phosphorylation at T220 significantly inhibits the calpain-induced hydrolysis of MAP2, a protection not observed with CaMKII-mediated phosphorylation (Johnson and Foley, 1993; Alexa et al., 1996; Alexa et al., 2002). Also, phosphorylation by PKA reduced binding to SH3 domain containing proteins, like Src and Grb2 (Lim and Halpain, 2000).

MAP2 phosphorylated by PKA from the brain can be dephosphorylated by calcineurin and protein phosphatase with Km values in the range of 1–3 μM and 1.6–2.7 μM, respectively (Goto et al., 1985; Yamamoto et al., 1988). PKA signaling also mediates dendritic remodeling. Activation of D1 dopamine receptors (D1Rs), which stimulate the cAMP-PKA pathway, increases MAP2 phosphorylation and correlates with decreased dendritic extension (Song et al., 2002).

### 4.5 Calcium/phospholipid-dependent protein kinase (PKC)

Protein kinase C (PKC) is a serine/threonine kinase widely distributed across various brain regions. It is activated by Ca^2+^ and phospholipids (Saito et al., 1988). PKC in brain tissue can be classified into three main subtypes *α*, *β* (I and II) and *γ*, with primary structures highly homologous and conserved. The γ subtype is localized in the brain and spinal cord uniquely (Domanska-Janik, 1996).

PKC was first identified as a kinase capable of phosphorylating MAP2 in rat brains, using two-dimensional gel electrophoresis (Rodnight et al., 1985). Subsequent studies revealed that PKC specifically phosphorylates serine residues on MAP2 and is capable of incorporating at least 15 moles of phosphate into MAP2 (Walaas and Nairn, 1989; Akiyama et al., 1986). The phosphorylation sites targeted by PKC are located within the tubulin-binding domain (S1703, S1711, and S1728 in mouse Map2b, homologous to S1702, S1710, and S1727 in human MAP2b) and the projection domain. The homologous phosphorylation sites have been validated in rat brain tissues (S1705, S1713, and S1730 in rat MAP2b) (Tsuyama et al., 1986; Ainsztein and Purich, 1994).

Functionally, PKC-mediated phosphorylation modulates the ability of MAP2 to interact with cytoskeletal components. Overall phosphorylation by PKC reduces both microtubule polymerization and actin cross-linking induced by MAP2, while phosphorylation at S1728 completely abolishes MAP2’s microtubule-binding ability (Hoshi et al., 1988; Ainsztein and Purich, 1994; Diaz-Nido et al., 1990). Furthermore, under pathological conditions such as hyperammonemia, PKC activity on MAP2 is significantly reduced, highlighting its regulatory importance in both normal and diseased states (Felipo et al., 1993).

### 4.6 Calcium/calmodulin-dependent protein kinase II (CAMKII)

CAMKII is a serine/threonine kinase activated upon binding of Ca^2+^/calmodulin. It is the most abundant post-synaptic domain protein. The three isoforms of CAMKII, CaMKIIβ, CAMKIIγ and CAMKIIδ, are more broadly distributed across the brain regions and cell types, whereas CaMKIIα is predominantly expressed in excitatory neurons and certain inhibitory neurons, such as Purkinje cells (Bayer et al., 1999). CAMKII is essential to synaptic plasticity, learning, and memory (Silva et al., 1992; Yasuda et al., 2022).

CaMKII phosphorylates MAP2 in the brain, incorporating up to 5 moles of phosphate into the protein (Vallano et al., 1986; Yamauchi and Fujisawa, 1982). Tryptic digestion and two-dimensional phosphopeptide mapping identified five major phosphopeptides targeted by CaMKII, four of which involve threonine residues. The remaining serine residue is shared as a phosphorylation site with PKA (Goldenring et al., 1985; Jefferson and Schulman, 1991). Interestingly, different studies using varied phosphorylation conditions and protease digestion have reported four overlapping phosphorylation sites between CaMKII and PKA (Walaas and Nairn, 1989; Jefferson and Schulman, 1991; Schulman, 1984). These phosphorylation sites identified *in vitro* have also been detected in rat brains and pancreatic cells (Yamamoto et al., 1983; Yamamoto et al., 1985; Krueger et al., 1997).

The functional consequences of MAP2 phosphorylation by CaMKII include inhibition of MAP2-induced microtubule assembly (Yamamoto et al., 1983). During development, MAP2 phosphorylation level decreases in proportion to the declining concentration of CaMKII in chicken brains, indicating a developmental regulation of MAP2 function by this kinase (Koszka et al., 1991). Additionally, the reversal of CaMKII-mediated phosphorylation by calcineurin and protein phosphatase C restores MAP2’s ability to promote microtubule assembly, underscoring the dynamic regulation of MAP2 phosphorylation and dephosphorylation in cellular processes (Goto et al., 1985; Yamamoto et al., 1988).

## 5 Mapping MAP2 phosphorylations

Whereas the above section focused on cataloging the effects of MAP2 phosphorylations by the upstream kinase tested, an alternative way to comprehend the effects of phosphorylation on MAP2 interactions is at the level of the individual site. In Supplementary Table 1 and Figure 1, we present a list of all currently identified MAP2 phosphosites, and where known, their impact on MAP2 function.

## 6 Conclusion

This review has summarized the effects of phosphorylation at various sites on MAP2 function, and looked to link these to the kinases that have been identified to date as able to phosphorylate MAP2 at those sites. As the predominant microtubule-associated protein in dendrites, MAP2 plays a crucial role in regulating cytoskeletal dynamics, serving as a scaffold protein for signaling pathways, and facilitating cargo transport within dendrites. MAP2 is highly phosphorylated *in vivo*, and this phosphorylation is tightly regulated during development. Dysregulation of MAP2 phosphorylation is associated with several neuropsychiatric and neurodegenerative disorders (for further discussion, please see DeGiosio et al., 2022).

Due to the intrinsically disordered nature of MAP2, phosphorylation flexibly influences MAP2 structure and thus its function, modulating its interactions with its many interacting proteins. However, existing studies of the effect of MAP2 phosphorylation have had several limitations. Most studies have focused on MAP2 binding to microtubules and actin, leaving effects on additional MAP2 protein interactions and resultant functions, such as regulation of trafficking, synaptic plasticity, or protein synthesis, largely unexplored. Advanced tools, such as phosphomimetic mutations in combination with proteomic techniques able to concurrently assess MAP2 interactions with multiple binding partners, provide valuable opportunities to address these gaps and identify the functional consequences of specific phosphorylation events in MAP2 in greater depth.

Additionally, the phosphorylation of MAP2 by kinases such as ERK2, CDK2, and PKA has been investigated extensively. However, systematic screening of MAP2 phosphorylations by individual kinases, for example by combining kinase exposure with phosphoenrichment and mass spectrometry, has not been widely employed, resulting in many phosphorylation sites potentially remaining unidentified. Moreover, approaches to identify kinase-specific phosphorylation sites *in vitro*, while valuable, do not fully replicate cellular phosphorylation dynamics. Future research should aim to overcome these limitations by combining *in vivo* approaches with high-resolution analytical techniques to achieve a comprehensive understanding of MAP2 phosphorylation and its functional implications.

A third limitation is the limited number of kinases studied in relation to MAP2 phosphorylation. While it is not feasible to experimentally investigate all potential kinases, this gap is being partially addressed through advances in bioinformatic methods that predict kinase-substrate interactions. Bioinformatic prediction of kinase activity is predicated on two components: (1) accurately and conclusively associating a kinase with a specific phosphosite, and (2) quality of assignment of the phosphosite to a specific amino acid within the protein. Within the last 20 years, advances in mass spectrometry-based proteomics have greatly improved both the specificity of phosphosite mapping and associating kinases with their substrates (DeMarco and Hall, 2023; Shuken, 2023). Several labs have endeavored to collate known kinase-phosphosite relationships across multiple species, and kinase substrate recognition sequences from the literature into publicly available databases, most notably PhosphoSite Plus (Supplementary Table 2) (Linding et al., 2008; Krug et al., 2019; Dinkel et al., 2011; Diella et al., 2004; Diella et al., 2008; Huang et al., 2018; Johnson et al., 2023; Poll et al., 2024; Yaron-Barir et al., 2024; Hornbeck et al., 2015). This has spurred the development of bioinformatics tools for inferring kinase activity based on the level of phosphorylation of its substrate or substrate recognition motif (Lai et al., 2012; Terfve et al., 2015; Wiredja et al., 2017; Casado et al., 2013).
